# News and Events of the Week

**Published:** 1910-03-26

**Authors:** 


### News and Events of the Week.

Sir Thomas Barlow, M.D., F.R.S., has been elected the President of the Royal College of Physicians of London in succession to Sir Richard Douglas Powell, M.D., F.R.C.P., who was elected in 1905.

Mr. William Ilbert Hancock, of 27 Queen Anne Street, Cavendish Square, London, W., F.R.C.S., formerly, senior assistant surgeon, pathologist and clinical assistant at the Central London Ophthalmic Hospital, who died January 20, aged 36, left estate valued at £17,460.

The thirteenth annual concert in aid of the Poor-law Officers' Benevolent and Orphan Fund will be held at the King's Hall, Holborn Restaurant, on Monday, April 11, 1910, under the chairmanship of H. Dawes, Esq. Several first-class and well-known artistes have been engaged to appear during the evening.

"Trips Through Spain" is a handsomely illustrated pamphlet compiled by M. J. Causse, and giving valuable information to intending tourists to the peninsula provinces. It is published under the auspices of the Orleans-Midi Railways Company, which issues special Easter trip tickets to tourists at considerably reduced rates.

At a meeting of the Honiton Board of Guardians serious reflections were made against one of the medical officers of health. It was stated that a poor man named William Henry Piper had been unable to obtain medical attention before his death. Dr. Bingley Pullin repudiated the accusation, and thereupon tendered his resignation. The resignation was accepted; a resolution that the doctor be asked to reconsider his decision being defeated by 16 votes to 12.

Professor Doyen will commence a special class of surgical anatomy and operative surgery in the large amphitheatre of the Faculty of Medicine at Paris on April 18 next. The course will comprise fifteen demonstrations and will be held daily, Sundays excepted, from 6 to 7 o'clock in the evening, and will be open gratuitously to all foreign graduate students who may care to attend. Dr. Doyen's well-known excellence as a post-graduate teacher is a sufficient guarantee for the success of the course.

At a specially convened and thoroughly representative meeting of the Rochdale and District Medical Society, held in the Wellington Hotel, Drake Street, Rochdale, on Thursday, February 24, 1910, the following resolutions were unanimously adopted :—1. "That this meeting, after hearing Dr. Harris's statement of his position as to the charges made against him by the plaintiff in the recent lunacy case, and having carefully considered the evidence, documentary and other, produced in support of such statement, unanimously and unhesitatingly declares its conviction of the unfounded nature of the charges made, and the absolute innocence of Dr. Harris with regard to them." 2. "That the members of this Society wish to offer to Dr. Harris their profound sympathy in the undeserved trouble brought upon him by no action of his, and to express their regret that, owing to legal technicalities, he is unable to make public (by reopening the trial) the real facts of the case." 3. "That copies of the foregoing resolutions be forwarded to the lay and medical Press."

Dr. H. E. Vincent, M.D., B.S., has been appointed Esquire Bedell at London University in succession to Dr. Kenrick, resigned.

The cordial thanks of the Senate of London University have been voted to Sir Francis Galton for a further donation of £500 to the Francis Galton Laboratory for the Study of National Eugenics during the year 1911-12.

Mr. H. R. Kenwood, C.M., M.B., Chadwick Professor of Hygiene, and Dr. W. J. R. Simpson, C.M.G., M.D., C.M., Professor of Hygiene at King's College, have been appointed to represent London University at the International Hygiene Exhibition to be held at Dresden in 1911.

The Preston Town Council has decided to confer the honorary freedom of the borough upon Dr. Robert Charles Brown, F.R.C.P., one of the oldest inhabitants of Preston. He has subscribed munificently to the new buildings of the Preston Royal Infirmary, of which he is consulting medical officer.

The *British Homœopathic Review* has issued its last number, after three years of existence. A committee has been formed to establish a new journal to carry on its work. We much regret the demise of our contemporary, which was always moderate and courteous in tone and very ably edited.

A Bill has been introduced into the Ontario Legislature which seeks to abolish compulsory vaccination. Municipal councils now have the right to pass by-laws to enforce compulsory vaccination. The Bill is to repeal the present Act, and to leave it to each person to decide for himself whether or not he shall be vaccinated.

The Right Hon. Lord Faber, D.L., J.P. (chairman of the *Yorkshire Post*), says that he will gladly preside at the seventy-first anniversary festival of the Newsvendors' Benevolent Institution, in response to the committee's unanimous wish. It is felt that this will emphasise the Society's national character more effectually than any other step.

The Swiss Federal Railway has ordered four Pullman coaches specially fitted for the transport of invalids. Each carriage, costing £2,400, will be divided into seven compartments, the centre being for the patients. There is to be an operating theatre for urgent cases and a pharmacy. The rest of the compartments will be for doctors, nurses, and patients' friends.

The Central Public House Trust Association has just issued for the use of motorists, cyclists, and other tourists and travellers a descriptive list of about 200 hotels and inns, situated throughout the kingdom, under the management of the Public House Trusts. Copies of the list, which gives the accommodation of each house, may be obtained gratis from the Secretary of the Association, 15 Dean's Yard, Westminster, S.W.

The international congress for the study of cancer, to be held in Paris under the patronage of the President in the first week of October, will be presided over, says the *Times*, by Professor Czerny and Professor Fibiger, of the

International Association. The other presidential members will be Professors Pierre Marie, Roswell Park, George Meyer, von Hausemann, Bouchard (of the Institute), Barrier, and Pierre Delbet; Drs. Ledoux-Lebard and Henri de Rothschild, and Professor Gabriel Petit.

The Research Defence Society has issued three new pamphlets in the "Truth about Vivisection" series. The first is a report of Dr. Ryle's speech at the inaugural meeting of the Brighton branch of the Society, in which the moral and ethical objections to vivisection are dealt with; the second is entitled "The National Welfare," and is by the Bishop of Winchester; while the third is a short exposition of the facts regarding the now notorious "Brown Dog."

The King has granted the following gentlemen his licence and authority to accept and wear the decorations stated against their names, which have been conferred on them in recognition of valuable services rendered :—Mr. A. A. W. P. Murison, M.B., of the Deaconesses' Hospital, Cairo, the Fourth Class of the Order of the Red Eagle, conferred upon him by the German Emperor, King of Prussia; and Mr. E. R. Wheeler, M.B., Ch.B., M.R.C.S., L.R.C.P., formerly Medical Adviser to the Kanagawa Prefecture, the Third Class of the Order of the Sacred Treasure, conferred upon him by the Emperor of Japan.

The result of the competition for the building of a new examination hall for the Royal Colleges of Physicians and Surgeons on the site of the four houses, Nos. 8, 9, 10 and 11 Queen Square, Bloomsbury, for which seven architects were invited to compete, was made known on March 16 at a joint meeting of the Royal Colleges of Physicians and Surgeons, when Mr. T. E. Collcutt, who was appointed assessor, awarded the first premium—namely, the appointment of architect for the new examination hall, to Mr. A. N. Prentice, F.R.I.B.A., of Hastings House, Norfolk Street, Strand. The three other premiums, £100, £75, and £50, were respectively awarded to Mr. Henry T. Hare, F.R.I.B.A., 13 Hart Street, Bloomsbury; Mr. E. Stanley Hall, 54 Bedford Square; and Mr. J. W. Simpson, F.R.I.B.A., 3 Verulam Buildings, Gray's Inn.

The second Municipal and Health Exhibition is to be held at the Royal Agricultural Hall, Islington, from May 7 to May 14. Such exhibitions are designed to make known the most efficient methods of street cleansing, the economic utilisation of refuse, the housing of the working-classes, and the construction of public buildings. Its promoters hope to achieve two objects: (1) To interest and instruct municipal officials; and (2) to educate the public in the municipal services upon which the rates are expended. The exhibits will be divided into two main classes—public works and internal administration—and they will illustrate the details of the construction of hospitals, asylums, town halls, public libraries, schools, workmen's dwellings, and the laying-out of cemeteries and public parks. There will also be exhibits in connection with public sanitation, municipal ambulances, municipal telephones, smoke abatement, water supply, and fire prevention.

At the Cardiff Assizes last week, before Mr. Justice Pickford, a civil action was concluded, in which Mr. Hubert Hope Thomas, L.R.C.P., and his partner, Mr. Robert James Farman, L.S.A., were sued for alleged negligent treatment by David Lewis, an engine-driver. The plaintiff stated that he was taken ill in May 1908, and that Mr. Farman diagnosed his complaint as indigestion, whereas he was really suffering from pleurisy. In consequence he became seriously ill, and had to be operated upon seven times. He had also to seek parish relief. Mr. J. D. Davies, M.B., who operated on the plaintiff, denied that he had urged the case on and had found the money. He admitted that the defendant, Mr. Thomas, had sued him for libel some years ago, and secured a verdict for £50. It was stated that Mr. Thomas was made a party to the action because it was he with whom the employés at the works had made their contract. Mr. Farman deposed that when he first saw Lewis he diagnosed his malady as influenza, and told him to keep to his bed. The defendant, Mr. H. H. Thomas, Mr. E. Le Cronier Lancaster, Dr. T. D. Griffiths, and other medical men were called for the defence. Mr. Francis Williams, K.C., for the defence, suggested that Mr. Davies was at the back of the case against his clients. The jury found for the plaintiff, to whom they awarded £125 damages.

The "Voyages d'Etudes Medicales," organised by Dr. de la Carriere, have for their object the promotion of knowledge derived from actual visits to the baths and thermal stations in France among medical men, both French and foreign practitioners. There are very many thermal and climatic "stations" in France, and the excursions are so arranged that members who participate in them can visit, under the guidance of experts, each in turn. The first excursion this year will start in September next, and a most interesting and exhaustive programme has been drawn up for this and subsequent tours. It may be mentioned that these excursions are limited to members of the profession and their wives and families, that special travelling facilities are offered, and that English medical men who in past years have taken part in these travels have been charmed with their outing, and speak in high terms of the excellent management and personal supervision of the executive committee which have contributed largely to the comfort of the professional tourists. Particulars of the tours, programmes, etc., may be obtained on application to Dr. Carron de la Carriere, President de la Société d'Hydrologie Medicale de Paris, 2 Rue Lincoln, Paris.

THE HOSPITAL. March 26, 1910.

